# Supplementary material for: WordCommentsAnalyzer: A windows software tool for qualitative research
Source: F1000Res. 2018 Sep 4;7:536. Originally published 2018 May 3. [Version 2] doi: 10.12688/f1000research.14819.2 (PMC6137411; doi:10.12688/f1000research.14819.2)
Supplement: Supplementary file 3 [file f1000research-7-17614-s0002.tgz › b2a79496-4ada-47c4-9682-a99ce867836d.zip!/Eight Iranian Health Journals/Yafteh _25.docx]

© Yafteh  . This work is licensed under CC BY 4.0 (https://creativecommons.org/licenses/by/4.0/).

Volume & Issue: دوره 14 - شماره 3

Title: بررسی ارتباط میزان سطوح سرمی بیومارکرهای استرس اکسیداتیو و پره اکلامپسی

http://yafte.lums.ac.ir/article-1-800-fa.html

مقدمه: پراکلامپسی یکی از علل مرگ و میر مادران و جنین می باشد. .هدف از مطالعه حاضر بررسی سطوح سرمی نشانگرهای زیستی پراکسیداسیون لیپیدی شامل ایزوپروستان F2α، مالون دی آلدئید و ظرفیت تام آنتی اکسیدانت در زنان باردار مبتلا به پره اکلا مپسی در مقایسه با زنان باردار نرمال بود. بحث و نتیجه‌گیری: از مطالعه حاضر می توان نتیجه گرفت که افزایش استرس اکسیداتیو و در نتیجه پراکسیداسیون لیپیدی می تواند به عنوان فاکتور مهم در پاتوفیزیولوژی پره اکلا مپسی مطرح باشد. یافته‌ها: سطوح سرمی ایزوپروستان F2α، مالون دی آلدئید و هموسیستئین در زنان باردار مبتلا به پره اکلا مپسی به طور معنی داری بالاتر از زنان باردار نرمال بود (05/0 > p). میانگین سطوح سرمی هر سه متغیر در زنان باردار مبتلا به پره اکلا مپسی شدید به طور معنی داری بالاتر از زنان باردار مبتلا به پره اکلا مپسی ملایم بود (05/0 > p). مواد و روش‌ها: یک مطالعه بررسی مقطعی طراحی شد. نمونه بیمار شامل 30 زن باردار مبتلا به پره اکلا مپسی و نمونه غیر بیمارشامل 30 زن باردار نرمال بود. نمونه ها با استفاده از روش نمونه گیری متوالی انتخاب شدند. میزان سطح سرمی مالون دی آلدئید با استفاده از روش رنگ سنجی اندازه گیری گردید. میزان سطوح سرمی ایزوپروستان F2α و هموسیستئین با استفاده از روش الایزا اندازه گیری شد. تجزیه و تحلیل آماری داده ها با استفاده از آزمون آماری من ویتنی انجام گردید. مقادیر 05/0 > p از نظر آماری معنی دار در نظر گرفته شد.

Title: حذف نیترات از آب با استفاده از کنسرسیوم باکتری‌های دنیتریفایر تثبیت شده بر روی کربن فعال در یک راکتور بستر شناور

http://yafte.lums.ac.ir/article-1-801-fa.html

مقدمه: یون‌های حاوی نیتروژن نظیر نیترات در برخی منابع آب در غلظت‌‏های بالا موجود می‌باشد. این آلاینده باعث ایجاد مشکلات بهداشتی مثل سندرم بچه آبی در نوزادان و احتمال تشکیل ترکیبات سرطان زا در آب و ایجاد سرطان دستگاه گوارش می‌شود لذا حذف آنها از منابع آب ضرورت دارد. دنیتریفیکاسیون بیولوژیکی یکی از روش‌های مؤثر در حذف نیترات می‌باشد که در این راستا می‌توان از راکتورهای بستر شناور استفاده نمود. هدف از این مطالعه ارزیابی راکتور بستر شناور با بستر کربن فعال در حذف نیترات از آب می‌باشد. مواد و روش‌ها: باکتری‌های دنیتریفایر جداسازی شده از فاضلاب کارخانه شیر خرم‌آباد بر روی کربن فعال گرانولی تثبیت شده و از آن به عنوان بستر در راکتور بستر شناور برای حذف نیترات استفاده شد. وضعیت تثبیت با تهیه تصویر میکروسکوپ الکترونی مورد بررسی قرار گرفت. حذف غلظت 200-50 میلی‌گرم بر لیتر نیترات از آب با زمان ماند 3-1 ساعت مورد ارزیابی قرار گرفت. این مطالعه تحت شرایط آنوکسیک انجام شد. همچنین اثرات شرایط بهره برداری نظیر زمان ماند هیدرولیکی، pH، غلظت اولیه نیترات، بار ورودی و نوع منبع کربن نیز مورد بررسی قرار گرفت. بحث و نتیجه‌گیری: این مطالعه نشان داد که کربن فعال گرانولی می‌تواند سایت مطلوبی را برای چسبیدن باکتری‌های دنیتریفایر تأمین نموده و به عنوان بستر، دنیتریفیکاسیون آب را در راکتور بستر شناور به خوبی انجام دهد. یافته‌ها: نتایج آزمایشات نشان داد که بیش از 94 درصد نیترات با زمان ماند 3-2 ساعت (غلظت نیترات ورودی 200-50 میلی‌گرم بر لیتر) حذف شده و با این راندمان تجمع نیتریت و آمونیاک وجود نداشت و غلظت نیترات، نیتریت و آمونیاک در آب خروجی در غلظت‌های ورودی 100-50 میلی‌گرم بر لیتر در حد استاندارد آب آشامیدنی بود. تا میزان بار ورودی kgNO3-N•m-3•day-1 4/2 کیفیت آب آشامیدنی برای نیترات موجود در آب تصفیه شده فراهم شد. همچنین حداکثر نرخ دنیتریفیکاسیون (kgNO3-N•m-3•day-145/3) برای میزان بار ورودی kgNO3-N•m-3•day-1 8/4 ایجاد گردید.

Title: بررسی میزان ناتوانی‌های بیماران پارکینسونی در بخش مغز و اعصاب بیمارستان شهدای عشایر و کلینیک های مغز و اعصاب خصوصی شهر خرم آباد در نیمه اول سال 1389

http://yafte.lums.ac.ir/article-1-802-fa.html

مقدمه: بیماری پارکینسون یک اختلال تحلیل برنده عصبی پیش رونده است و شامل علائم حرکتی و غیرحرکتی متعددی می‌ باشد که هر کدام به نوبه خود سبب ناتوانی و اختلال عملکرد در سطح گسترده در بیماران می‌شود. این مطالعه با هدف بررسی ناتوانی های بیماران پارکینسونی انجام شد. مواد و روش‌ها: مطالعه به صورت سری-موردها بود. افسردگی بر اساس معیار DSM-IV ، میزان اختلالات شناختی بر اساس آزمون کوتاه وضعیت ذهنی MMSE و ناتوانی بیماران بر اساس معیارهوئن و یارتشخیص داده شد. اختلال عملکرد بویایی با چهار ماده آب، گلاب، توتون و قهوه تشخیص داده شد. بحث و نتیجه‌گیری: این بررسی نشان داد اختلالات حرکتی و غیرحرکتی با شیوع بالایی در بیماران پارکینسونی وجود دارد. ناتوانی در انجام فعالیت‌ها تحت تأثیر فاکتورهایی چون سن، طول مدت درمان و طول مدت بیماری است. بنابراین تشخیص بیماری و درمان مناسب آن قبل از بروز ناتوانی ها باید مورد توجه قرار گیرد. یافته‌ها: در مجموع 79 بیمار مورد مطالعه، بیماری در مردان سه برابر زنان، بیشترین رده سنی درگیر 79-70 سال و شیوع افسردگی و اختلالات شناختی6/45 و 9/32 درصد بود. شیوع حملات روشن- خاموش، حملات زمین خوردن و اختلال بویایی به ترتیب 5/21،9/32 و 4/68 درصد و فراوانی بیماران در مرحله سوم نسبت به سایر مراحل بیماری بیشتر بود. بین طول مدت بیماری و نیز طول مدت درمان و میزان ناتوانی ارتباط معنی داری مشاهده شد. ارتباط بین افسردگی و میزان ناتوانی و نیز شدت بیماری با 006/0= Pمعنی دار بود.

Title: فراوانی هلیکوباکترپیلوری در اطفال مبتلا به اسهال حاد و ارتباط آن با بروز اسهال

http://yafte.lums.ac.ir/article-1-803-fa.html

مقدمه: هلیکوباکترپیلوری از دسته باسیل‌های گرم منفی است که منجر به ایجاد گاستریت‌های هیستولوژیک کاملاً مشخصی گردیده و در ایجاد سرطان‌های معده نیز نقش آن معلوم گردیده است. میزان فراوانی این باکتری در جوامع و گروه‌های سنی مختلف متفاوت است، اما اکثریت افراد در اوایل زندگی آلوده به این باکتری می‌شوند و منجر به ایجاد علائم و بیماری‌های مختلفی مثل گاستریت ، دل درد ، کم خونی و... می‌گردد. هدف از انجام این مطالعه، تعیین فراوانی هلیکوباکترپیلوری در اطفال مبتلا به اسهال حاد بستری در بیمارستان شهید مدنی خرم آباد در تابستان 1389 بود. بحث و نتیجه‌گیری: 7/16% از اطفال مبتلا به اسهال حاد از لحاظ تست سرولوژیک هلیکوباکتر پیلوری مثبت شدند و بروز اسهال درکودکان را ضرورتاً نمی‌توان از علائم همیشگی ابتلا به هلیکوباکتر پیلوری دانست . یافته‌ها: در کل 240 کودک مبتلا به گاستروانتریت مورد بررسی قرار گرفتند که8/60% آنها مذکر و 2/39%آنها مؤنث بودند و در مجموع 7/16% آنها از لحاظ تست سرولوژیک هلیکوباکترپیلوری مثبت شدند. اکثریت موارد مثبت متعلق به جنس مذکر(60%) و گروه سنی زیر 2 سال (45%) بوده و اکثرا ً(5/72%) ساکن شهر و مصرف کننده آب لوله‌کشی بهداشتی بودند. در بین موارد مثبت فقط 5/17% در منزل حیوان خانگی نگهداری می‌کردند که اکثریت با ماکیان بوده و 5/67% آنها سابقه بستری در مراکز درمانی را ذکر نمودند و شایعترین علت بستری، عفونت‌های تنفسی بود. مواد و روش‌ها: نوع این مطالعه توصیفی ـ مقطعی و روش نمونه گیری به صورت سرشماری بود. جمعیت مورد مطالعه شامل کلیه اطفالی بودند که در تابستان سال 1389 با تشخیص گاستروانتریت در بیمارستان شهید مدنی خرم‌آباد بستری شدند. البته شرط ورود به مطالعه این بود که اطفال بستری شده، قبلاً تحت درمان ضدهلیکوباکترپیلوری قرار نگرفته باشند. متغیرهای مورد بررسی شامل جنسیت، گروه سنی، محل سکونت، نوع آب آشامیدنی غالب، نگهداری حیوان خانگی و سابقه بستری در مراکز درمانی بود. اطلاعات مربوط به بیماران از طریق پرسشنامه جمع‌آوری گردید و در عین حال نمونه‌های سرمی بیماران، تهیه شده و در آزمایشگاه مرجع از طریق تست ELIZA در رابطه با وجود آنتی بادی (IgG) ضد هلیکوباکترپیلوری مورد آزمایش قرار گرفتند.

Title: نقش تعادل میان KIRهای مهاری و فعال کنندگی در تعیین استعداد ابتلا به اسپوندیلیت انکیلوزان

http://yafte.lums.ac.ir/article-1-804-fa.html

مقدمه: اسپوندیلیت انکیلوزان(AS) یک بیماری پیشرونده و ناتوان‌کننده است که تقریباً 9/0% افراد را در سراسر دنیا منتلا می‌سازد. تاکنون مکانیسم دقیق شروع و پیشرفت AS شناخته نشده است. ارتباط میان HLA-B27 و AS به‌عنوان قوی‌ترین ارتباط بین یک مولکول آنتی‌ژن لکوسیتی انسان (HLA) کلاس I و بیماری باقی است. علی‌رغم تحقیقات وسیع، نقش پاتوژنیک این ژن و محصول آن هنوز حل نشده است. همچنین ژن‌هایی غیر از HLA-B27وجود دارند که به‌نظر می‌رسد در اتیولوژی بیماری دخیل باشند. مجموعه ژنی پذیرنده‌ شبه ایمونوگلبولینی سلول کشنده (KIR) بر روی کروموزوم q13.419 در کمپلکس پذیرنده لکوسیت قرار گرفته است. ژن‌های KIR گروهی از مولکول‌ها را کد می‌کنند که بر روی سلول‌های کشنده طبیعی (NK) و در برخی سلول‌های T بیان می‌شوند. پروتئین‌های KIR به‌عنوان پذیرنده‌هایی عمل می‌کنند که مولکول‌های HLA کلاس I را شناسایی می‌کنند و به‌طور مستقیم در فعالیت و مهار سلول‌های NK دخیل می‌باشند. KIRها و لیگاندهای HLA کلاس I آنها در پاتوژنز انواع مختلف بیماری‌های خودایمنی مشارکت دارند. عدم تعادل KIRهای مهاری و فعال‌کنندگی، عامل کلیدی است که می‌تواند پاتوژنز AS را تحت تأثیر قرار ‌دهد. با این وجود نقش تعادل میان KIRهای مهاری و فعال کنندگی در تعیین استعداد ابتلا به AS یک موضوع قابل بحث است. این مقاله مروری خصوصیات اصلی این ژن ها را خلاصه کرده و بحث می کند که چگونه ممکن است آنها در پاتوژنز AS درگیر باشند.

Title: بررسی تاٌثیر استفاده از بانداژ فشاری در کم کردن عوارض موضعی و عوارض ناشی از بی‌حرکتی در بیماران بعد از عمل آنژیوگرافی عروق کرونر در بیمارستان های قلب شهید رجایی تهران و شهیدمدنی خرم‌آباد

http://yafte.lums.ac.ir/article-1-805-fa.html

بحث و نتیجه‌گیری: بنابراین با توجه به افزایش راحتی بیمار و تأخیر در بروز خونریزی، بانداژ فشاری می‌تواند به طور معمول در کنترل خونریزی و کبودی این بیماران بعد از آنژیوگرافی کرونری مورد استفاده قرار گیرد. مواد و روش‌ها: این مطالعه، تحقیقی نیمه‌تجربی است که در بخش‌های CCU و جراحی بیمارستان قلب شهید رجایی تهران و شهید مدنی خرم‌آباد انجام شده است. تعداد 100 بیمار که تحت عمل آنژیوگرافی قرار گرفته‌اند، به صورت تصادفی انتخاب شدند و پس از اعمال فشار دستی به ناحیه شریان فمورال، 50 نفر آنها در گروه کیسه شن و 50 نفر آنها در گروه بانداژ فشاری به طور تصادفی قرار گرفتند. یافته‌ها: بیمارانی که در گروه کیسه شن بودند، مقدار بروز خونریزی در آنها بعد از بیرون کشیدن سوند، بالاتر بود و خیلی سریع‌تر از بیمارانی که در گروه بانداژ فشاری بودند خونریزی می‌کردند (05/0P<). بروز و وسعت کبودی در هر دو گروه یکسان بود ولی بیمارانی که در گروه کیسه شن بودند، میزان بالاتری از درد پشت و درد کشاله ران را تجربه کردند. مقدمه: عروق کرونر قلب، مسئول خون‌رسانی به عضله قلب می‌باشند و چنانچه در اثر گرفتگی رگ به علت پلاک چربی مسدود شده باشند، بایستی بیمار تحت آنژیوگرافی عروق کرونر قرار گیرد تا میزان تنگی معلوم شود و برای این منظور از شریان رانی چپ استفاده می‌شود. هدف از این تحقیق، بررسی تأثیر بانداژ فشاری در کم کردن خونریزی، کبودی، درد کمر و درد کشاله ران در بیمارانی است که تحت عمل آنژیوگرافی عروق کرونر قرار می‌گیرند.

Title: مقایسه دو روش درمانی جااندازی بسته و پین گذاری و جااندازی باز درشکستگی سوپراکندیلار نوع III استخوان بازو در کودکان زیر ده سال

http://yafte.lums.ac.ir/article-1-806-fa.html

مقدمه: شکستگی استخوان بازو یکی از شکستگی‌های شایع در کودکان می‌باشد. دو سوم بچه‌هایی که به خاطر جراحات بازو بستری می‌شوند دچار شکستگی سوپراکندیلارهستند. هدف از انجام این مطالعه بررسی دو روش درمانی "جااندازی بسته و پین‌گذاری از راه پوست " و "جااندازی باز و تثبیت داخلی " درشکستگی سوپراکندیلار نوع III استخوان بازو در کودکان زیر ده سال بود. بحث و نتیجه‌گیری: با توجه به نتایج بدست آمده از این مطالعه و همچنین سایر مطالعات مشابه، روش جراحی بسته در شکستگی استخوان بازو از نظر عوارض پس از عمل، هزینه و مدت بستری در بیمارستان ارجحیت داشته و کارآیی بیشتری در درمان شکستگی استخوان سوپراکوندیلار دارد. یافته‌ها: بین دو گروه از نظر جنس، سن، محل سکونت شهری و روستایی، سمت شکستگی و شکستگی دست غالب اختلاف معنی‌داری وجود نداشت. در یک هفته بعد از عمل، 3 نفر(66/6%) از بیماران عمل شده با روش باز و 1 نفر(22/2%) از بیماران عمل شده به روش بسته مبتلا به عفونت شدند(011/0=P). میانگین دامنه حرکت مفصل در هفته هشتم بعد از عمل در بیماران عمل شده به روش باز 9/10 ± 3/118 و در بیماران عمل شده به روش بسته 5/10 ± 5/131 درجه بود (001/0>P). میانگین مدت عمل جراحی و هزینه عمل در بیماران عمل شده به روش جراحی باز به طور معنی‌داری بیشتر از بیماران عمل شده به روش بسته بود (001/0>P). مواد و روش‌ها: دراین مطالعه کارازمایی بالینی 90 کودک مبتلا به شکستگی استخوان سوپرا کندیلار نوع III (بر اساس طبقه‌بندی گارتلند) مراجعه کننده به مرکز آموزشی درمانی آیت ا ... کاشانی شهرکرد در سال 1389 به روش آسان انتخاب و به کمک جدول اعداد تصادفی به 2 گروه تقسیم شدند. بیماران گروه 1 با جااندازی بسته و پین‌گذاری از روی پوست و با کنترل C-arm و بیماران گروه 2 با روش جا اندازی باز و تثبیت داخلی شکستگی با استفاده از پین و با دسترسی خلفی تحت عمل جراحی قرار گرفتند. اطلاعات بیماران از نظر مدت زمان عمل جراحی، بررسی رادیوگرافی، عفونت محل زخم، هزینه‌های درمانی، میزان محدوده حرکتی مفصل آرنج در هفته هشتم بعداز عمل, جمع آوری و به کمک آزمون‌های آماری t-test و chi square مورد تجزیه و تحلیل قرارگرفت.

Title: مقایسه نظریه خاص ذهن سوء استفاده کنندگان جنسی از زنان با افراد عادی

http://yafte.lums.ac.ir/article-1-807-fa.html

مقدمه: هدف پژوهش حاضر بررسی مقایسه ای نظریه خاص ذهن سوء استفاده کنندگان جنسی از زنان با افراد عادی بود. بحث و نتیجه‌گیری: مشکلات سوء استفاده‌کنندگان جنسی در حوزه‌های صمیمت، همدلی و تحریفات شناختی از نقص در نظریه ذهن ناشی می‌گردد که با افزایش آنها می‌توان مشکلات سوء استفاده‌کنندگان جنسی را تقلیل داد. یافته‌ها: نتایج نشان داد که سؤ استفاده‌کنندگان جنسی از زنان نسبت به گروه عادی در نظریه خاص ذهن تفاوت معنی‌داری دارند. یعنی سوء استفاده‌کنندگان جنسی از زنان در نظریه خاص ذهن از نقص بیشتری برخوردارند. مواد و روش‌ها: این پژوهش از نوع مورد- شاهدی بود. جامعه آماری این پژوهش کلیه سوء استفاده‌کنندگان جنسی زندانی رده سنی 20 تا 35 ساله طی سال‌های 89-1388 شهر ارومیه بودند که از این جامعه تعداد 30 سوء استفاده کننده جنسی زنان با روش نمونه‌گیری در دسترس انتخاب شدند. همچنین برای انتخاب گروه نمونه عادی تعداد 30 نفر که از لحاظ ویژگی‌های سنی، جنسی و تحصیلات با گروه سوء استفاده‌کننده جنسی همتا شده بودند، انتخاب شدند. برای گردآوری داده‌ها از آزمون چشم بزرگسالان برای ارزیابی نظریه عام استفاده شد. از سویی برای تجزیه و تحلیل داده‌ها، از روش‌های آماری توصیفی و آزمون t استفاده شد.

Title: بررسی اثر دوز پایین آسپرین ( mg/kg 7/5) بر بافت تخمدان در زمان لانه‌گزینی در موش سفید آزمایشگاهی نژاد NMRI

http://yafte.lums.ac.ir/article-1-808-fa.html

مقدمه: آسپرین یک داروی ضد التهاب غیراستروئیدی است که با تأثیر بر روی پروستاگلاندین‌ها در تکامل فولیکول ها و ساختار جسم زرد دخالت دارد. با توجه به این موضوع بررسی تأثیر دوز پایین آسپرین در زمان لانه گزینی ضروری به نظر می رسد که در تحقیق حاضر به آن پرداخته شده است. بحث و نتیجه‌گیری: دوز پایین آسپرین(mg/kg5/7( در زمان لانه گزینی با افزایش تعداد جسم زرد بر روند حاملگی اثر مثبت داشته و باعث دوام جنین می‌شود، همچنین با افزایش مقاطع عروقی باعث خون‌رسانی بالا و افزایش کارایی تخمدان می شود. یافته‌ها: وزن تخمدان در گروه کنترل gr077/0±3/1 و در گروه تجربی g‏r 24/0±55/3 و نیز حجم تخمدان در گروه کنترلmm3 19/0± 70/8 و در گروه تجربی mm3 17/0±20/9 بود. تعداد جسم زرد در گروه کنترل 15/0±2/6 و در گروه تجربی 15/0±21/12 شمارش شد)در هر سه مورد تفاوت معنی دار مشاهده شد). در خصوص مطالعات کیفی مربوط به مقاطع عروقی به نظر می‌رسد تعداد مقاطع عروقی در گروه تجربی نسبت به گروه کنترل افزایش یافته است. مواد و روش‌ها: موش‌های ماده نژاد NMRI به دو گروه کنترل و تجربی تقسیم شده و در هر دو گروه بارداری کاذب القا شد، گروه تجربی تا روز 5/4 تحت تزریق با آسپرین قرار گرفت، سپس در روز 5/4 تخمدان‌ها خارج شده، ابتدا حجم و وزن آنها اندازه گیری شد، سپس برش های سریالی تهیه و رنگ‌آمیزی به روش H&E انجام گرفت.

Title: دیدگاه متخصصان سلامت در مورد معیارهای لازم برای ارزیابی عملکرد مدیران بیمارستان‌ها

http://yafte.lums.ac.ir/article-1-809-fa.html

مقدمه: به منظور عملکرد بهتر مدیران بیمارستان ها نیاز به یک ارزیابی از عملکرد آنها بر اساس معیارهای مشخص وجود دارد. اما متاسفأنه هیچ سیستم ارزیابی عملکرد رسمی و نظام مندی برای ارزیابی مدیران به کار گرفته نشده است. بنابراین هدف پژوهش حاضر تعیین معیارهای ملموس و مناسب جهت ارزیابی عملکرد مدیران بیمارستان ها با استفاده از نظر متخصصین می باشد. بحث و نتیجه‌گیری: با توجه به سابقه اجرایی افراد شرکت کننده ، نظرات آنها گام مؤثری برای شناسایی معیارهای تأثیرگذار و مهم برای تدوین ابزاری برای ارزیابی عملکرد مدیران بیمارستان ها خواهد بود. ولی بدیهی است به منظور تدوین چنین ابزاری باید علاوه بر استخراج نظرات متخصصین و مطالعات انجام شده در سایر کشورها، یافته ها با توجه به شرایط بیمارستان های کشور بومی شوند. یافته‌ها: هفت درون مایه اصلی در خصوص معیارهای لازم جهت ارزیابی عملکرد مدیران بیمارستان ها از بررسی نظر متخصصین بدست آمد، این درون مایه ها عبارتند از مهارت های مربوط به برنامه ریزی، سازماندهی و مدیریت عملکرد کارکنان، هدایت و رهبری، مدیریت اطلاعات، مدیریت منابع، حاکمیت بالینی و شاخص های عملکردی. مواد و روش‌ها: این مطالعه یک پژوهش کیفی بود که با روش تحلیل درون مایه ای انجام شد. با استفاده از پرسشنامه نیمه ساختار یافته با 2 نفر از صاحبنظران نظام سلامت مصاحبه شد و 20 نفر در جلسات بحث گروهی متمرکز شرکت کردند. بعد از اتمام هر مصاحبه و بحث گروهی، برداشت ها با شرکت کنندگان چک گردید. متن مصاحبه های اولیه همزمان توسط دو نفر از اعضای هیئت علمی تحلیل شد و سپس تلفیق گشت.

Title: تاثیر عوامل اصلی اکولوژیک بر درصد بازده اسانس درختچه مورد (Myrtus communis L) در رویشگاه های مختلف جنگلی استان لرستان

http://yafte.lums.ac.ir/article-1-810-fa.html

مقدمه: مورد Myrtus communis درختچه همیشه سبزی است که از خانواده Myrtaceae می‌باشد. در نظر گرفتن ویژگی‌های محل رویش و موقعیت گیاه در طبیعت از عمده عواملی است که می‌تواند بر میزان اسانس و مواد موثره گیاه تاثیر زیادی داشته باشد. بحث و نتیجه‌گیری: رویشگاه کرکی کمترین میزان تولید اسانس را در بین رویشگاه های مورد استان لرستان به خود اختصاص داده است. همچنین در مجموع جهت جنوب غربی با جهت‌های شمالی و جنوبی به لحاظ میزان اسانس تفاوت معنی‌داری نشان داد. یافته‌ها: نتایج حاصل از ضریب همبستگی پیرسون، وجود ارتباط مستقیم معنی‌دار بین ارتفاع از سطح دریا و درصد بازده اسانس (358/0r= و049/0p= ) و همچنین ارتباط معکوس بین میزان سدیم خاک و درصد بازده اسانس (405/0r = و 027/0p = ) را نشان داد. بین درصد بازده اسانس در رویشگاه‌های مختلف و همچنین جهات جغرافیایی مختلف اختلاف معنی‌داری مشاهده گردید. مواد و روش‌ها: به منظور بررسی تاثیر عوامل اصلی اکولوژیکی موثر بر میزان اسانس درختچه مورد، ازچندین رویشگاه اصلی مورد دراستان لرستان نمونه برداری به عمل آورده و از هر منطقه 40 گرم برگ خشک اسانس گیری شد و درصد بازده اسانس برگ های مناطق مختلف به دست آمد. علاوه بر آن مولفه های اصلی فیزیوگرافیک هر کدام از رویشگاه ها (شیب، جهت دامنه، ارتفاع از سطح دریا) نیز اندازه گیری شد. از بین عوامل اکولوژیک قابل اندازه گیری نیز عناصر سدیم، پتاسیم، منیزیوم و کلسیم و میزان اسیدیته و شوری خاک مورد اندازه گیری قرار گرفت

Title: امفالوسل و قلب بیرون از قفسه سینه درنوزاد با Pentalogy of Cantrell

http://yafte.lums.ac.ir/article-1-811-fa.html

بحث و نتیجه‌گیری: در تمام موارد ابتلا به امفالوسل بررسی از نظر وجود پنتالوژی کانترل و آنومالی همراه توصیه می‌شود. معرفی مورد: در این مقاله نوزاد مبتلا به امفالوسل و قلب بیرون از قفسه سینه (ectopia cordis) معرفی می‌شود . مقدمه: پنتالوژی کانترلPentalogy of Cantrell) ) اختلال مادرزادی بسیار نادری است که با نواقص شدید قفسه سینه و شکم شامل بیرون زدگی احشاء از محل بندناف (امفالوسل)، نقص قسمت پایینی جناغ سینه ، نقص قسمت جلویی دیافراگم، نقص قسمت جلویی پریکارد، قلب بیرون از قفسه سینه (ectopia cordis) تشخیص داده می‌شود. پاتوژنز این ناهنجاری ناشناخته است . نقایص تکاملی لایه مزدرمی رویان در روزهای 18-14 پس از لقاح را عامل مؤثر در بروز این ناهنجاری می‌دانند.
